# Integrative proteomics and metabolomics approach to identify the key roles of icariin-mediated protective effects against cyclophosphamide-induced spermatogenesis dysfunction in mice

**DOI:** 10.3389/fphar.2022.1040544

**Published:** 2022-12-14

**Authors:** Chunyan Fang, Yulong Ye, Fang Yang, Fangyue Wang, Yifeng Shen, Degui Chang, Yaodong You

**Affiliations:** ^1^ TCM Regulating Metabolic Diseases Key Laboratory of Sichuan Province, Hospital of Chengdu University of Traditional Chinese Medicine, Chengdu, China; ^2^ Tea Research Institute, Sichuan Academy of Agricultural Sciences, Chengdu, China; ^3^ Hospital of Chengdu University of Traditional Chinese Medicine, Chengdu, China

**Keywords:** cyclophosphamide, spermatogenesis dysfunction, icariin, proteomic, metabolomic

## Abstract

The alkylating antineoplastic agent cyclophosphamide (CP) is known to be toxic to the male reproductive system, but there are no effective prevention or treatment options. The flavonoid icariin (ICA), which is used in Chinese medicine, has been shown to have a number of biological functions, including testicular protection. The current study looked into the protective effects of ICA in preventing CP-induced spermatogenesis dysfunction. The current study looked into the role of ICA in preventing testicular dysfunction caused by CP. For 5 days, healthy adult mice were given saline or a single dose of CP (50 mg/kg) intraperitoneally (i.p). For the next 30 days, mice were given ICA (80 mg/kg) by gavage. Animals were euthanized 12 h after receiving ICA, and testes were removed for biochemical, histopathological, sperm evaluation, and transmission electron microscope analysis (TEM). We also investigated the potential biological effects of ICA on CP-induced spermatogenesis dysfunction in mice using an integrated proteomic and metabolomic approach. The levels of 8309 proteins and 600 metabolites were measured. The majority of the differential proteins and metabolites were found to be enriched in a variety of metabolic pathways, including the PI3K-Akt signaling pathway, necroptosis, the mTOR signaling pathway, glycerophospholipid metabolism, and ABC transporters, implying that ICA may have molecular mechanisms that contribute to CP-induced spermatogenesis dysfunction in the testis. Taken together, these findings show that ICA effectively reduces testis injury, implying that ICA may have a role in male infertility preservation.

## Introduction

Cancer is one of the deadly diseases of the world (Fusco et al., 2021). With the advancement of diagnosis and treatment technology, the survival rate of many cancers has increased dramatically in recent years. Cancer has been treated in a variety of ways, including surgery, chemotherapy, and drugs. CP is one of the most effective and widely used anticancer drugs ever developed (Balow et al., 1987). It is commonly used to treat sarcomas, leukemias, breast and prostate cancers, neuroblastoma, lymphoma, and multiple myeloma (Ludeman, 1999; Pagnoux, 2016). It is also used to treat granulomatosis with polyangiitis, nephrotic syndrome, organ transplant rejection, and a variety of other diseases (Kern and Kehrer, 2002; Ahmed et al., 2015). However, the gonadal failure caused by CP is a major issue, particularly in males.

The primary pharmacological activity of CP, a cyclic phosphoramide ester, is to destroy the ability of all cells within its reach to grow and reproduce by cross-linking DNA strands (Vernet et al., 2004). Because of the presence of uninterrupted cell division, the reproductive system is extremely sensitive to CP (Trasler and Robaire, 1988). CP causes testicular and epididymal tissue damage in male subjects, resulting in low testosterone levels, oligospermia, and asthenospermia (Ghobadi et al., 2017a). CP administration in adult male rats decreases testicular weight and modifies the expression of stress response genes in the testis, according to animal studies (Aguilar-Mahecha et al., 2002). Exposure to CP has been shown to destroy genomic structure and increase lipid preoxidation (Ghobadi et al., 2017b). However, our understanding of the molecular mechanisms underlying CP toxicity in the testis remains limited.

ICA is a primary bioactive substance derived from the Chinese medicinal herb *Epimedium*. Previous research has shown that ICA has a diverse set of biological and pharmacological properties, including anti-inflammation, anti-tumor, anti-depression, anti-aging, and reproductive system protection (Kong et al., 2015). By regulating the expression of steroidogenic acute regulatory protein (StAR) and peripheral type benzodiazepine receptor, ICA can significantly increase sperm counts and testosterone levels in the male reproductive system (PBR). In adult male mice, ICA could improve sexual function by regulating the PI3K/AKT/eNOS/NO signaling pathway (Derosa et al., 2018). Furthermore, animal studies demonstrated that ICA administration prevented testicular dysfunction caused by di (2-ethylhexyl) phthalate (DEHP) by lowering ROS levels in Leydig cells (Sun et al., 2019). These findings suggest that ICA may play a role in male fertility preservation.

In this paper, we used a CP-induced spermatogenesis dysfunction mouse model to investigate the protective role of ICA on spermatogenesis dysfunction. Because little is known about the effects of ICA on spermatogenesis dysfunction, we investigated the proteome and metabolome of testis exposed to CP and/or treated with ICA to investigate potential mechanisms.

## Materials and methods

### Animals and treatments

Adult male Kunming mice weighing 18–22 g were acquired from Chengdu Dossy Experimental Animals CO., Ltd. in Chengdu, China, and raised at the Animal Center of Chengdu University of Traditional Chinese Medicine, Chengdu, China. With forced air circulation, a 12-h light/dark cycle, a controlled room temperature of 22°C, and fresh food and water, all animals were housed in an animal cabinet. All animal protocols were authorized by Chengdu University of Traditional Chinese Medicine’s Animal Care Committee and adhered to the rules and standards in effect at the time by our Institutional Animal Ethics Committee (Ethics committee approval document number: 20211309A).

All of the mice were randomly assigned to one of three groups (*n* = 10): normal control (Ctrl group), CP only (CP group), or CP and ICA (ICA group). The control mice were given intraperitoneal injections of normal saline (NS) on a daily basis. For 5 days, the male mice (model) were injected with 50 mg/kg/d CP (*via* NS as the carrier) to induce testis injury. NS was administered to the Ctrl and CP groups over the next 30 days. The ICA group received an 80 mg/kg/d dose of ICA (Sichuan ChemConst Biotechnology Co., Ltd., China, lot number CONST210202, ICA>98%) intravenously for 30 days. All mice were euthanized by exsanguination under urethane anesthesia after treatment. The testis and epididymis were then removed and weighed, and the gonadal index was calculated using the following formula: Testicular or epididymal weight (mg)/body weight (g) = gonadal index (mg/g). Each mouse’s left epididymis was cut into pieces for sperm collection. The quality of the sperm was assessed and recorded using a computer-aided semen analyser (Beijing Suijia Medical Instrument) and manually. The left testis was severed. Half of the testis was fixed in 4% paraformaldehyde for histologic examination, while the other half was fixed in 3% glutaraldehyde for transmission electron microscopy (TEM). The appropriate testes were kept at −80°C until use.

### Histologic evaluation

Light microscopy was used to examine paraffin-embedded testicular tissues that had been sectioned to a thickness of 5 μm, stained with hematoxylin and eosin (H&E), and observed. A seminiferous tubule is scored according to its histological characteristics with Johnsen’s scoring system. There is no seminiferous epithelium in Score 1, there are no germinal cells, only Sertoli cells are present in Score 2, only spermatogonia are present in Score 3, and hardly any spermatocytes are present in Score 4; the score 5 indicates that there are many spermatocytes; the score 6 indicates that there are few early spermatids; the score 7 indicates that there are many early spermatids; A score of 8 indicates spermatogenesis is impaired, there are fewer late spermatids, disorganized epithelium, and a score of 10 indicates spermatogenesis is complete. (Takahashi et al., 2021).

### Transmission electron microscopy

New gonadal tissues were prepended with 3% glutaraldehyde, then postfixed in 1% osmium tetroxide, dehydrated in series acetone, and embedded after being infiltrated in Epox 812 for an extended period of time. Icluding sections were stained with methylene blue, and ultrathin sections were stained with uranyl acetate and lead citrate. The sections were examined using a JEM-1400-FLASH Transmission Electron Microscopy (TEM).

### Data independent acquisition mode for proteomic analysis

SDS lysis buffer (8 M urea, 1% SDS), containing an appropriate protease inhibitor, was used to extract proteins. The protein was quantified using the BCA method. We start taking an equivalent amount of each sample pool’s trypsin-digested peptides and suction dry them before rehydrating them in UPLC loading buffer (Phase A: 2% acetonitrile, pH 10; Phase B: 80% acetonitrile, pH 10). The blended peptides were separated using Vanquish Flex binary UHPLC chromatography (Thermo, United States) with an Acquity UPLC BEH C18 Column (1.7 μm, 2.1 mm 150 mm, Waters, United States). From the mixed sample, 28 fractions were extracted. In brief, the C18-reversed phase column (75 μm × 25 cm, Thermo, United States) was equilibrated with solvents A (2% ACN, 0.1% formic acid) and solvents B (80% ACN, 0.1% formic acid). At a flow rate of 300 nL/min, the tryptic peptides were separated.

To automatically switch between full scan MS and MS/MS acquisition, the Q Exactive HF-X instrument was configured to data-dependent acquisition mode (DDA). To automatically switch between full scan MS and MS/MS acquisition, the Q Exactive HF-X instrument was configured to data-independent acquisition mode (DIA). All precursor ions were then chosen for fragmentation by higher-energy collision dissociation in the collision cell following a survey of full scan MS spectra (m/z 300–1500). DIA was carried out using a variable isolation window, with each window overlapping by 1 m/z, for a total of 40 windows. High level of confidence peptides were employed for protein identification documents by setting a target false discovery rate (FDR) threshold of 1% at the peptide level. For protein identification, only proteins with at least one distinct peptide were used. Spectronaut (Biognosys AG, Version 14) was used to analyze the DIA data files, with the retention time prediction type set to dynamic iRT. Spectronaut 14 determined data extraction based on extensive mass calibration. Spectronaut uses iRT calibration and gradient stability to constantly find the best extraction window.

### Metabolomic analysis

The metabolites were isolated by using a 400 μl methanol:water (4:1, v/v) olution and 0.02 mg/mL L-2-chlorophenylalanin as an endogenous control. The samples were reconstitute in a 100 μl loading solution of acetonitrile:water (1:1, v/v) for UHPLC-MS/MS analysis. In order to create a pooled quality control sample (QC), equal volumes of each sample were combined as part of the system conditioning and quality control process. Similar to how the analytic samples were evaluated and disposed of, the QC samples were also. It helped to represent the whole sample set and was injected at regular intervals (every eight samples) to check on the stability of the analysis.

Thermo Fisher Scientific’s UHPLC-Q Exactive HF-X system was performed using for LC-MS analysis. Thermo UHPLC-Q Exactive HF-X Mass Spectrometer with an electrospray ionization (ESI) source that could operate in positive or negative ion mode was used to acquire the mass spectrometric data. Data was gathered using the Data Dependent Acquisition (DDA) technique. The mass range used for the detection was 70–1050 m/z.

Following the completion of the mass spectrometry detection, the raw LC/MS data is preprocessed by Progenesis QI (Waters Corporation, Milford, United States) software, and it exports a three-dimensional data matrix in CSV format. The data matrix was then deredundant and peak pooled. HMDB (http://www.hmdb.ca/), Metlin (https://metlin.scripps.edu/), and Majorbio Database were used to search for and identify metabolites at the same time. Following the database search, the data is uploaded to the Majorbio cloud platform (https://cloud.majorbio.com) for analysis. At least 80% of the samples had metabolic characteristics that were maintained. Following filtering, each metabolic characteristic was standardized by sum, and minimum metabolite values were imputed for particular samples whose metabolite levels were below the lower threshold for quantification. In order to minimize errors brought on by sample preparation and instrument instability, the response strength of the sample mass spectrum peaks was standardized using the sum normalization method, and the normalized data matrix was created. Simultaneously, variables exhibiting RSDs greater than 30% of QC samples were eliminated, and the final data matrix for further investigation was generated using log10 logarithmization.

### Bioinformatics analysis

For detecting correlation between testis tissue at different altitudes, R packages were used to perform principal component analysis on quantified proteins. The Q value (FDR) threshold for the precursor and protein levels was set at 1%. For quantification, the intensities of the six peptides with the highest intensities were chosen. Differentially expressed proteins (DEPs) were identified when the fold change (FC) was greater than 1.2 or less than 0.83 and *p*-value <0.05 (Student’s t test). Additionally, the Mfuzz tool was utilized to classify the DEPs based on their differential expression level administration of CP and ICA (Futschik and Carlisle, 2005). Principal component analysis (PCA) and orthogonal least partial squares discriminant analysis (OPLS-DA), as well as 7-cycle interactive validation, were carried out using the R package ropls (Version 1.6.2). Differentially expressed metabolites were found when FC > 1 or FC < 1, VIP >1 (OPLS-DA) and *p*-value <0.05 (Student’s t test) were present. To determine the functions of the proteins and metabolites, they were annotated against Gene Ontology (GO) and the Kyoto Encyclopedia of Genes and Genomes (KEGG). In order to identify whether a collection of metabolites occurred in a function node, enrichment analysis was frequently performed. A single metabolite annotation analysis was supposed to lead to a group of metabolite annotation analyses. Scipy stats (Python packages) (https://docs.scipy.org/doc/scipy/) was used to find statistically substantially enriched pathways using Fisher’s exact test.

### RNA extraction and RT-PCR

The MicroElute Total RNA kit (Omega) was used to isolate total RNA from testes. To synthesize cDNA, we used the PrimeScriptTM RT Reagent Kit with gDNA Eraser (TaKaRa) with 1 ug total RNA. An analyzetikjena qTOWER3G system and a Yeasen SYBR Green Master Mix were used for RT-PCR. The Glyceraldehyde-3-phosphate dehydrogenase (Gapdh) was used as an internal reference. The 2^−ΔΔCT^ method was used to calculate the effects of each experiment independently, with three biological replicates. A list of primers is provided in Supplementary Table S12.

### Statistical analysis

A statistical analysis was carried out using the GraphPad Prism 7 (GraphPad Software). The data are presented as the mean ± standard error (SE). We used Student’s t-test to compare two sets of data. The statistical significance level was set at *p* < 0.05; *p < 0.05, **p < 0.01, and ***p < 0.001.

## Results

### ICA administration protected the reproduction organ in the CP-induced spermatogenesis dysfunction mice model

**FIGURE 1:**
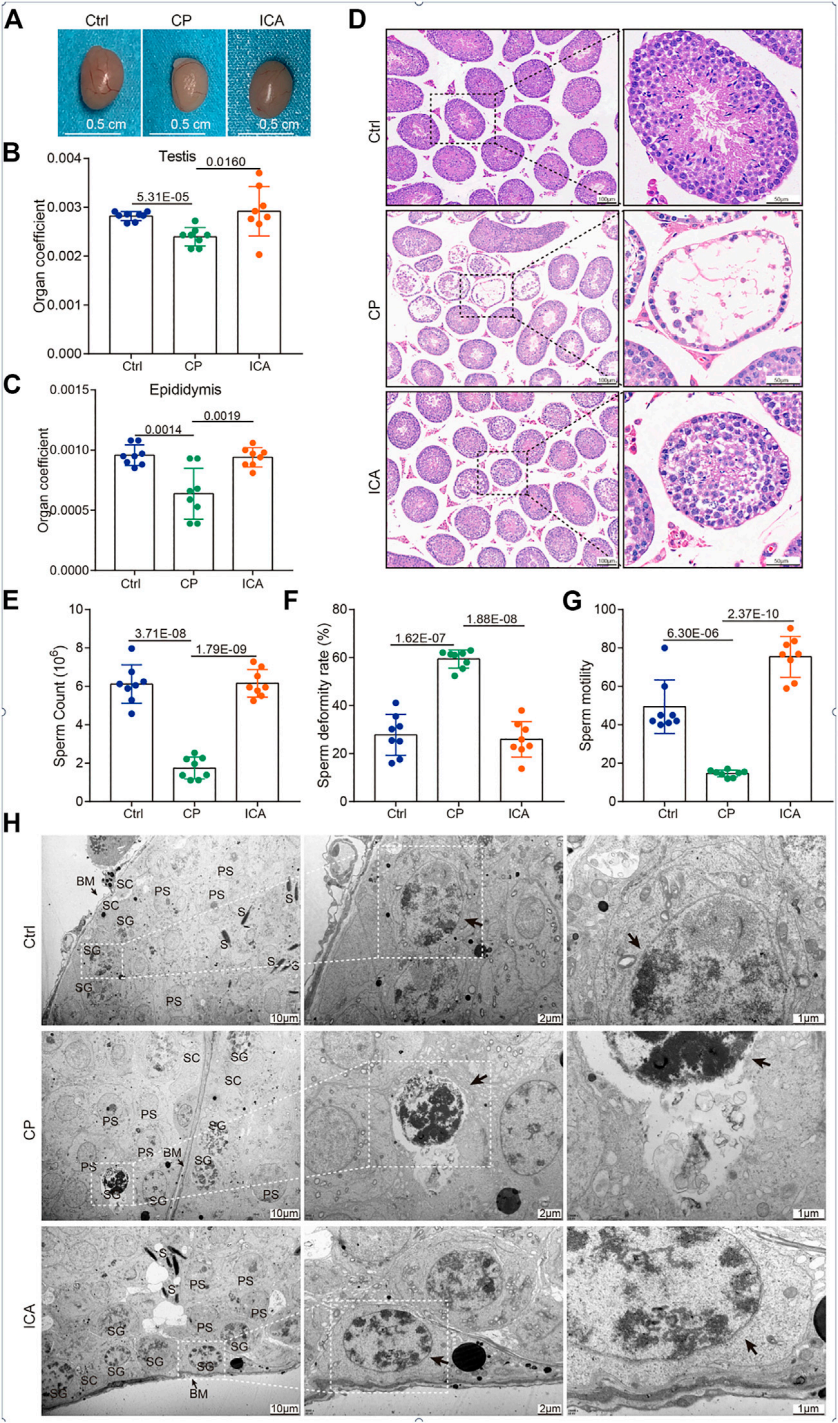
Protective effects of ICA on the CP-induced testicular damage mice model. **(A)** Gross morphology of the testis. **(B)** The effect of ICA on the testis coefficient. **(C)** The effect of ICA on the epididymis coefficient. **(D)** Representative images of H&E staining (100 × and 400 ×) of the testis from different groups as indicated (*n* = 8). **(E)** ICA increases sperm counts induced by CP in mice. **(F)** ICA attenuates sperm deformity in male mice. **(G)** ICA improved sperm motility. *p* < 0.05 was considered statistical difference (*n* = 8). **(H)** The ultrastructural changes of testis were observed under TEM from different groups (*n* = 3). BM, basement membrane; SG, spermatogonia; S, spermatid; SC, Sertoli cells; PS, primary spermatocyte. The black arrow indicates spermatogonia.

To explore whether ICA could protect the reproduction system in male mice, we first characterized the testis size from different groups. The testis size of the CP group was significantly smaller than that of the control mice, as shown in Figure 1A. After 30 days of ICA treatment (80 mg/kg/d), the ICA group’s testis size was significantly larger than the CP group’s. The reproductive organ cofficient (testis or epididymis) of ICA-treated male mice was significantly higher when compared to CP-exposed male mice (Figures 1B,C). Seminiferous tubules in the CP group had a normal architecture with a circular outline or elliptocytosis, and spermatocytes had a normal structure with no obvious abnormality, according to histological analyses (Figure 1D). In the CP group, however, the testes showed a disruption of the framework of the seminiferous tubules as well as a large number of vacuoles (Figure 1D). After CP administration at a dose of 80 mg/kg, testicular tissue appears restored with normal cell morphology of seminiferous tubules and spermatocytes when compared to the CP group (Figure 1D). Further, the Johnsen scores of testes in the ICA-treated mice were significantly higher than those in the CP group (Supplementary Figure S1).

Furthermore, the significant difference in sperm count, sperm deformity, and sperm motility between the CP-treated and other groups demonstrated harm to spermatogenesis. Sperm count and motility were significantly higher in the ICA-treated (80 mg/kg) mice than in the CP-exposed mice (Figures 1E,F). Furthermore, the ICA group had significantly lower sperm deformity rates than the CP group (Figure 1G). In addition, CP causes ultrastructural changes in the testis. In the control group, the spermatogonia were flat oval, and the spermatogonia nuclei were abundant but evenly distributed (Figure 1H). However, CP administration resulted in spermatogonial damage, including severely condensed nuclei and little dark cytoplasm. The spermatogenic epithelium of the testis in the ICA group was similar to that in the control group, according to TEM (Figure 1H). These findings suggested that mice given ICA (80 mg/kg) could significantly reduce the sperm deformity caused by CP.

### Proteomic and metabolomic profiling of mouse testis

**FIGURE 2:**
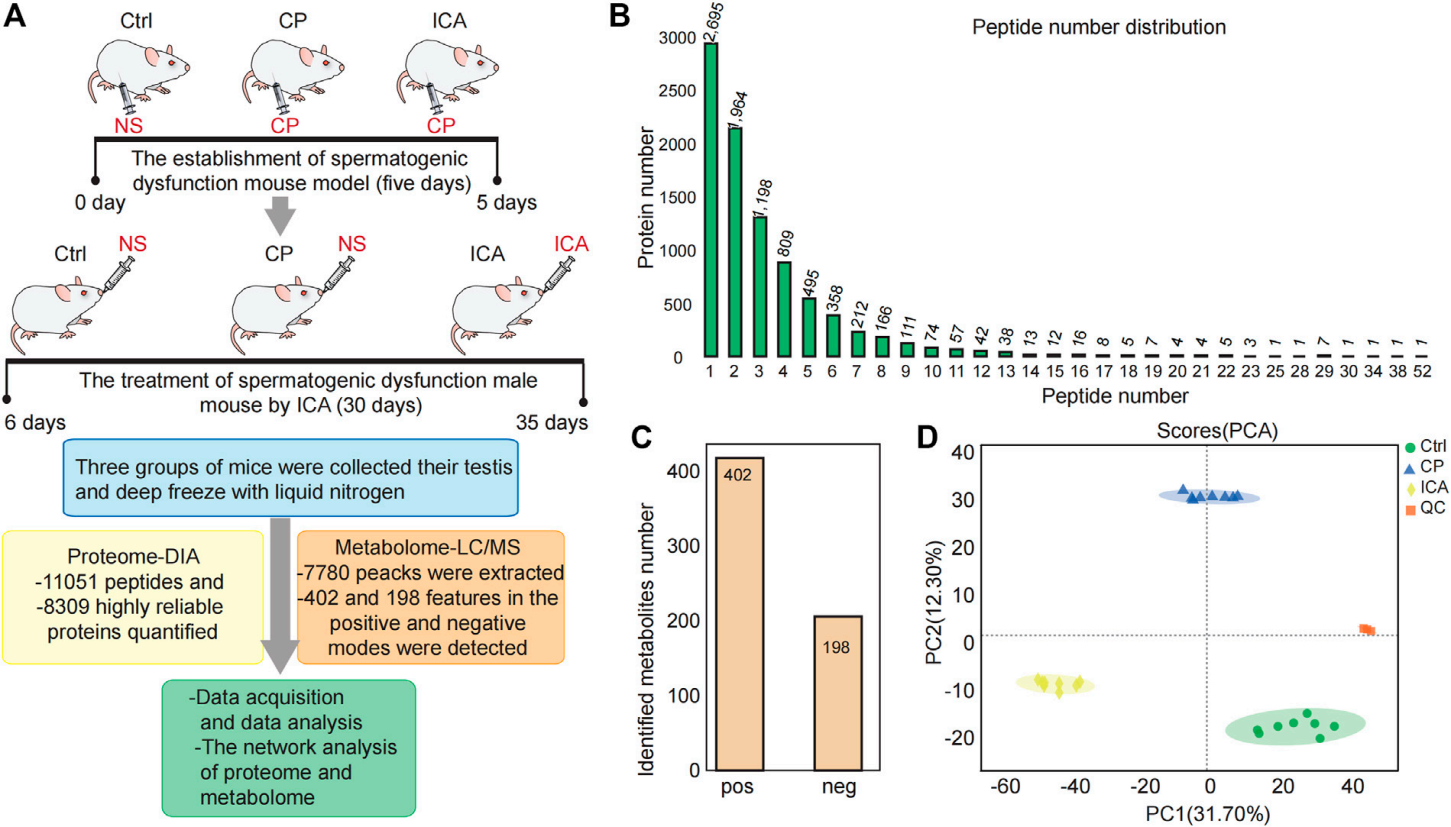
Overview of the testis proteomics and metabolimics analysis. **(A)** Experiment Workflow. Three groups—The Ctrl group injected with Normal saline (NS) (*n* = 10), Mod group injected with cyclophosphamide (CP) (*n* = 10), ICA group injected with cyclophosphamide in building models of spermatogenesis dysfunction (*n* = 10), were used in this study. The ICA treatment group to 80 mg/(kg.d) ICA irrigation stomach for 30 days. The normal and the model groups each received an equivalent NS. The testis tissue was obtained for omic platforms applied and analysis. **(B)** Distribution of peptide number in three groups. **(C)** Number of characterized metabolites in the Ctrl, CP and ICA samples. **(D)** Principal component analysis (PCA) of the quantified metabolites for each sample.

We performed proteomic and metabolomic analyses of mouse testis tissue to better understand the molecular mechanisms underlying ICA’s therapeutic effects on CP-induced spermiogenesis damage (Figure 2A). In this study, the data-independent acquisition mass spectrometry (DIA-MS) identification technology was used. The samples from three different groups could be well separated using principal component analysis (PCA) (Supplementary Figure S2). The DIA quantitative proteomics analysis identified 11,051 peptides (Figure 2B) and 8309 highly reliable proteins in all 9 samples (three groups with three technical replicates each) (Figure 2B). 5614 (60%) of the 8309 proteins had two or more unique peptides (Figure 2B).

The raw metabolomic data were collected using LC/MS methods. There were 7780 peacks extracted in total. In all 24 samples (three groups with eight technical replicates each), we found 402 and 198 features in the positive and negative modes, respectively (Figure 2C). To assess system stability at the global metabolomic level, unsupervised PCA was used. The PCA plot showed that the control testis were clearly separated from the CP and ICA testis, indicating that the LC-MS system was stable (Figure 2D).

### Identification of proteome changes in Ctrl, CP, and ICA mice testis

**FIGURE 3:**
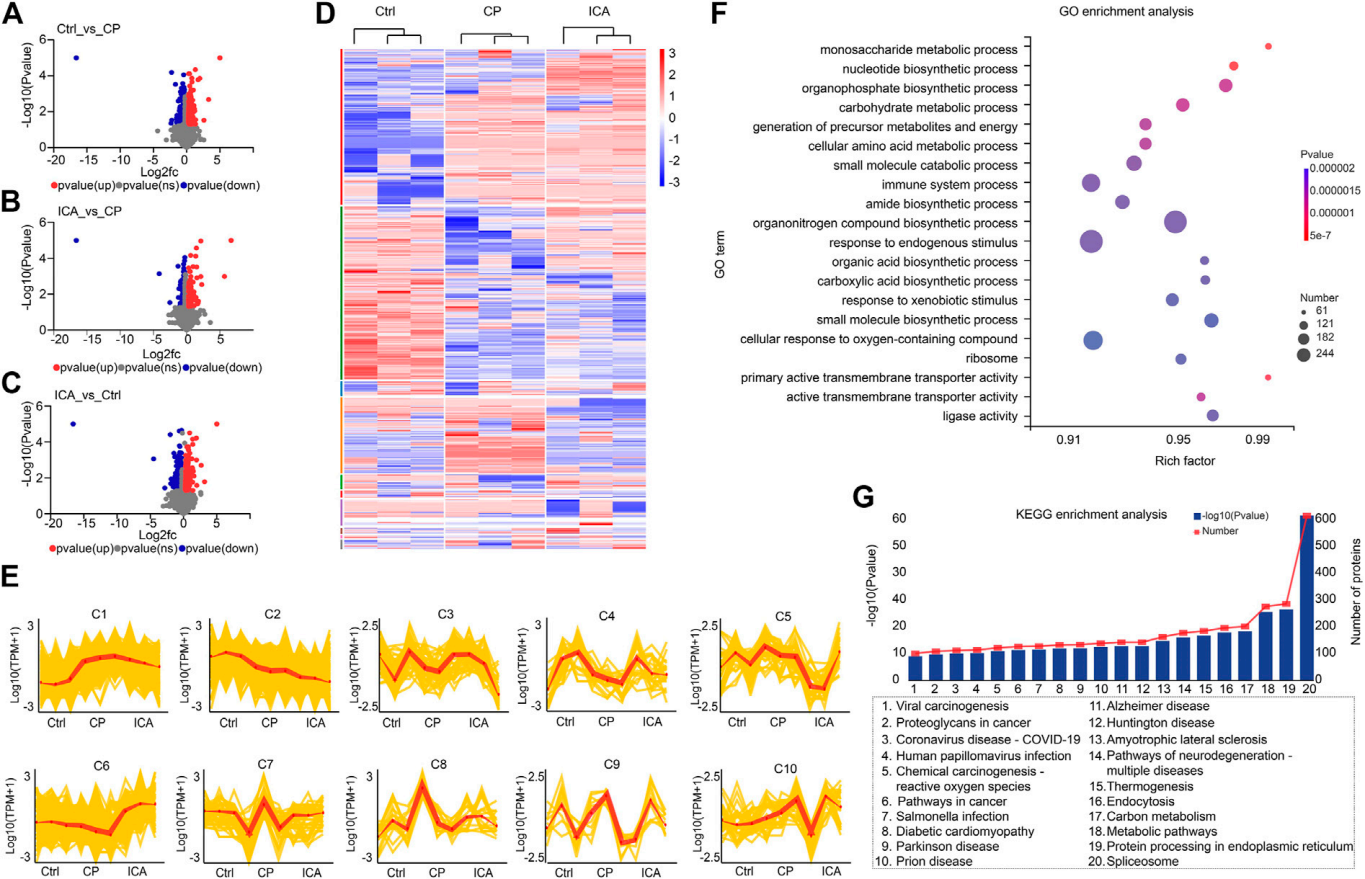
Quantitative proteomic analysis of testis in Ctrl, CP, and ICA mice. **(A)** Volcano plot of DEPs in destructive testis induced by CP compared to the control testis. **(B)** Volcano plot showing DEPs in testis treated with ICA compared to the destructive testis. **(C)** Volcano plot comparing DEPs between ICA-treated testis and control testis. **(D)** Dysregulated proteins in different groups shown as a heatmap. **(E)** The expression trends of proteins in ten sub-clusters. **(F)** The top 20 enriched GO terms of dysregulated proteins. The *x*-axis indicates the enrichment factor (RichFactor) which represents the number of DEPs annotated for each pathway divided by the number of all identified proteins annotated to the same pathway. A greater RichFactor indicates greater enrichment. The *Y*-axis shows the match ratio of GO terms. Bubble size represents the number of differentially expressed proteins in the pathway, and color indicates enrichment significance. **(G)** Top 20 dysregulated protein KEGG pathways.

To identify differentially expressed proteins (DEPs) in the testis between the control group (Ctrl), cyclophosphamide-induced spermatogenesis dysfunction group (CP), and icariin group (ICA), proteins with an adjusted *p*-value of 0.05, FC > 1.2 (up-regulated), or FC < 0.83 (down-regulated) were considered significantly DEPs. When the CP group was compared to the Ctrl group, 468 DEPs were found, including 174 up-regulated proteins and 294 down-regulated proteins (Figure 3A). While 351 DEPs were chosen, 174 up-regulated and 177 down-regulated proteins were found in ICA samples compared to CP samples (Figure 3B). Proteomic data revealed that 574 proteins were differentially expressed in the ICA group versus the Ctrl group, with 222 up-regulated proteins and 352 down-regulated proteins (Figure 3C). Then, we cross-matched these DEPs from the Ctrl/CP, ICA/CP, and Ctrl/ICA groups and discovered 61 overlapped proteins (Supplementary Figure S3), indicating that ICA may play a role in spermatogenesis dysfunction treatment by regulating these 61 proteins.

We performed a hierarchical cluster analysis based on three groups to systematically invistate the protein expression changes among the three groups (Figure 3D). According to the findings, all DEPs were classified into ten clusters with distinct expression trends (Figure 3E). Among the ten clusters, proteins in clusters 4 (C4), C6, and C7 were down-regulated in the CP group when compared to the Ctrl group, but up-regulated in the ICA group when compared to the CP group. A total of 262, 23, and 74 proteins were classified as C4, C6, and C7, respectively (Supplementary Table S1). Given that the ICA could increase protein expression in these three clusters, we hypothesized that forced exoression of these three protein clusters could compensate for spermatogenesis damage in the testis.

GO and KEGG pathway enrichment analyses were performed to better understand the function of these DEPs. The biological process (BPmost)’s significantly enriched GO terms were monosaccharide metabolic process, nucleotide biosynthetic process, organophosphate biosynthetic process, and carbohydrate metabolic process (Figure 3F; Supplementary Table S2). The main enriched GO terms of molecular function were primary active transmembrane transporter activity, active transmembrane transporter activity, ligase activity, and ATP-dependent activity (MF). Furthermore, the ribosome, cell junction, synapse, and catalytic complex were the main representative GO terms of the cellular component (CC). The KEGG pathway enrichment results revealed that metabolic pathways, neurodegeneration-multiple diseases pathways, Amyotrophic lateral sclerosis pathways, and so on were the most significantly enriched (Figure 3G; Supplementary Table S3). These findings suggest that metabolic and disease-related pathways are important for cariin-mediated protection against spermatogenesis dysfunction in mice.

### Screening and characterization of differential matabilites in testis

**FIGURE 4:**
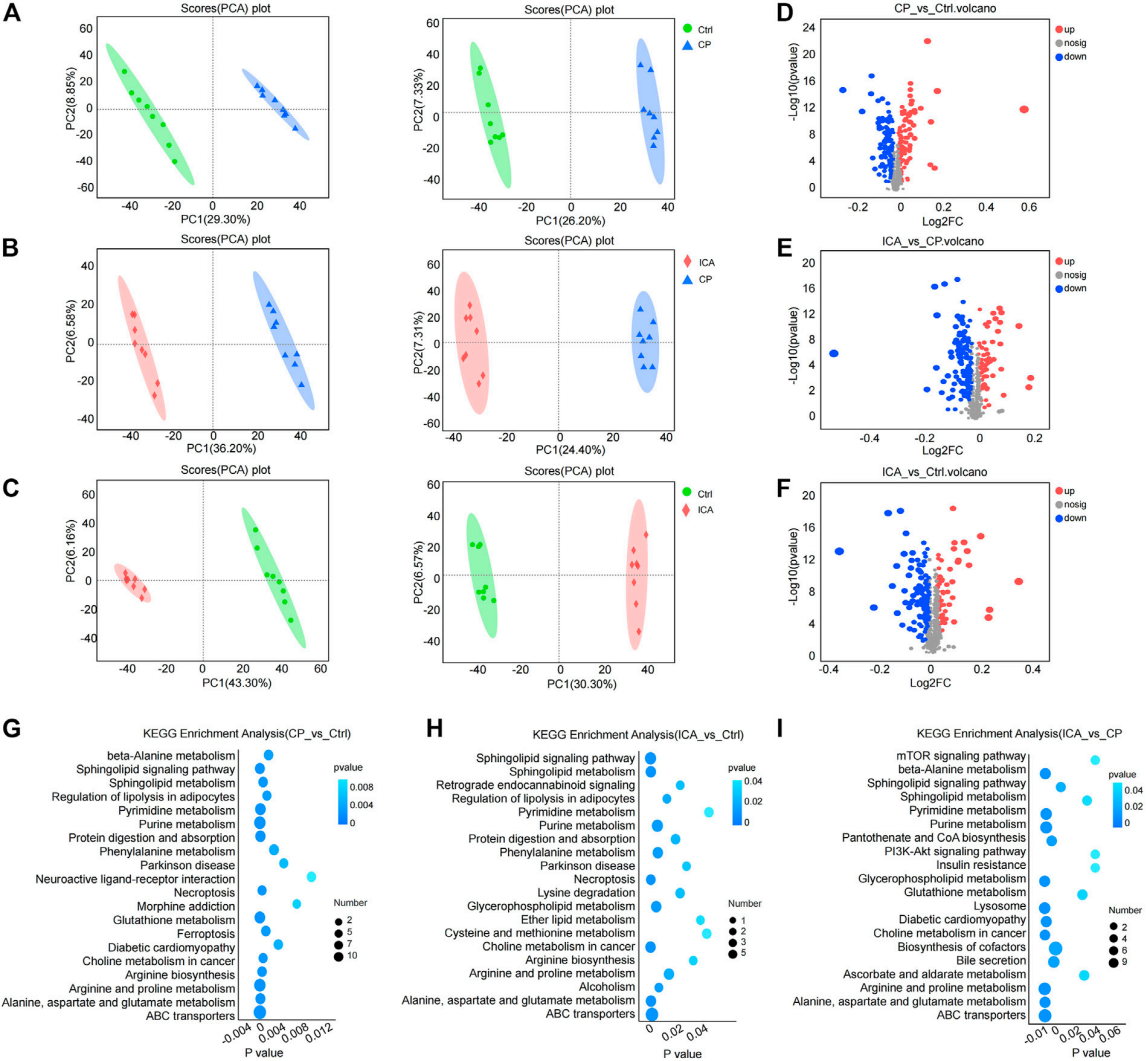
Metabolomics analyses revealed metabolic changes in ICA treatment of dysfunctional testis induced by CP. **(A)** PCA plots of “Ctrl vs. CP” in positive (left) and negative (right) ion scanning mode. **(B)** PCA plots of “CP vs. ICA” in positive (left) and negative (right) ion scanning mode. **(C)** PCA plots of “Ctrl vs. ICA” in positive (left) and negative (right) ion scanning mode. **(D)** Volcano map of “Ctrl” vs. CP”. **(E)** A volcano map comparing “CP” and “ICA”. **(F)** An overview of the volcano map of “Ctrl” versus “ICA”. **(G)** The enriched top 20 KEGG pathways of “Ctrl vs. CP”. **(H)** The enriched top 20 KEGG pathways of “CP vs. ICA”. **(I)** The enriched top 20 KEGG pathways of “Ctrl vs. ICA”.

The levels of testis metabolites were measured using LC-MS/MS to further evaluate the treatment of ICA in CP-induced spermatogenesis dysfunction mice. Eight biological replicates were investigated. PCA was used to cluster “Ctrl vs. CP”, “CP vs. ICA”, and “Ctrl vs. ICA”. The PCA revealed a clear distinction between the three groups (Figures 4A–C). Furthermore, the OPLS-DA score plot revealed a significant separation effect between groups (Supplementary Figure S4).

VIP and *p* values are the most commonly used criteria for accumulating potential metabolites. Significant metabolic perturbations between the two groups were defined as VIP >1 from OPLS-DA and *p*-value <0.05 from Student’s t-test. Furthermore, fold-change >1 indicated that the metabolite was trending upward, whereas fold-change <1 indicated a trending downward. As a result, 231 (“CP vs. Ctrl”) metabolites were obtained, with 118 increased and 113 decreased (Figure 4D; Supplementary Table S4); ICA exposure resulted in 208 (“ICA vs. CP”) altered metabolites, with 67 increased and 141 decreased metabolites (Figure 4E; Supplementary Table S5). Furthermore, 67 up-regulated metabolites and 131 down-regulated metabolites were detected in the testis following the ICA challenge compared to the Ctrl group (Figure 4F; Supplementary Table S6).

The KEGG pathways were enriched in the differential metabolites of the three comparison groups. According to the KEGG analysis, CP influenced Alanine, Aspartate, and Glutathione metabolism, Beta-Alanine metabolism, Arginine and Proline metabolism, and Sphingolipid metabolism (Figure 4G; Supplementary Table S7). The metabolism of Sphingolipids, Alanine, Aspartate, and Glutamate, Glutathione, Amino sugar and nucleotide sugar, Arginine, and Proline were modulated by ICA treatment (Figure 4H; Supplementary Table S8). Furthermore, CP conduction was found to regulate Sphingolipid metabolism, Glutathione metabolism, Alanine, aspartate, and glutamate metabolism, Glycerolipid metabolism, and Arginine and proline metabolism (Figure 4I; Supplementary Table S9). These findings show that the metabilisms of Alanine, Aspartate and glutamate, and Arginine and proline are associated with ICA’s protective effect on spermatogenesis impairment in the testis.

### Interative analysis of proteomics and metabonomics datasets derived from tesis of different groups

**FIGURE 5:**
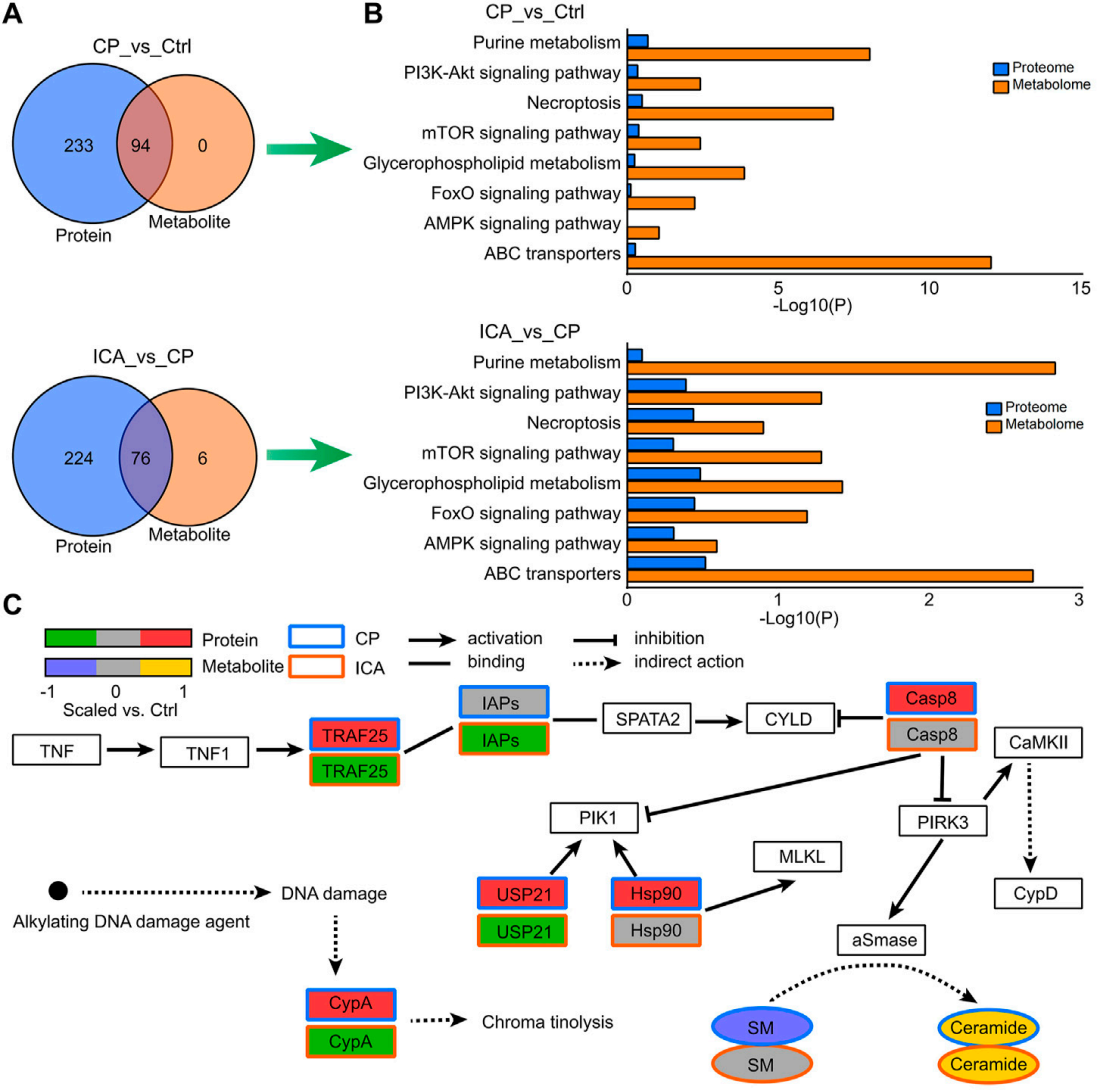
Enriched pathways from the combined analysis of proteomics and metabolomics data. **(A)** Venn diagrams illustrating the number or protein and metabolite pathways significantly changed in “Ctrl vs. CP” and “CP vs. ICA”. **(B)** The enriched KEGG pathways in “Ctrl vs. CP” and “CP vs. ICA”, which were expressed at both the protein and metabolite level. **(C)** Protein and metabolite modifications in the necroptosis pathway.

The KEGG pathways of identified dysregulated proteins and metabolites were used to draw Venn diagrams, which revealed that there were 94, 76, and 79 commonly enriched pathways from the Ctrl vs. CP and ICA vs. CP, respectively (Figure 5A). The most significantly enriched pathways were selected as the key pathway of ICA in the treatment of spermatogenesis dysfunction in mice, including PI3K-Akt signaling pathway, Necroptosis, mTOR signaling pathway, Glycerophospholipid metabolism, and ABC transporters (Figure 5B). In Figure 5C, as an example of pathway level changes, we show protein and metabolite changes in the Necroptosis pathway. The pathway analysis revealed that CP induced the up-regulation of expression of TRAF25, CypA, IAPs, Casp8, USP21, Hsp90, CaMKII, and CypD on testis in mice. Nevertheless, ICA could inhibit expression of TRAF25, FAF, USP21, ESCRT-III, and CypD to protect testis tissue. Moreover, qRT-PCR results confirmed that the mRNA levels of these genes significantly increased after exposure to CP and reduced after treating with ICA (Supplementary Figure S5). This indicate ICA administration effectively reduces Necroptosis expression to improve the testis morphology of CP-induced injury in testis tissue.

Supplementary Data of Supplementary Tables S10, S11 contain all of the regulated pathways in “CP vs. Ctrl” and “ICA vs. CP” at the protein and metabolite levels. One of the significant protein pathways in both “CP vs. Ctrl” (*p* = 0.0110) and “ICA vs. CP” (*p* = 0.0159) was the Protein digestion and absorption, highting the central role of this protein metabolism pathway (Supplementary Tables S10, S11). The key metabolic pathways such as Glutathione metabolism (“CP vs. Ctrl”: *p* = 0; “ICA vs. CP”: *p* = 1 × 10^–4^), Arginine and proline metabolism (“CP vs. Ctrl”: *p* = 0; “ICA vs. CP”: *p* = 1 × 10^–4^), ABC transporters (“CP vs. Ctrl”: *p* = 0; “ICA vs. CP”: *p* = 0.0021), and Choline metabolism in cancer (“CP vs. Ctrl”: *p* = 6 × 10^–4^; “ICA vs. CP”: *p* = 3 × 10^–4^) were also almost the top ranked pathways.

## Discussion

CP was a traditional drug that was primarily used in the treatment of various diseases, including cancer (Feng et al., 1972). However, the extreme cytotoxicity and side effects, such as reproductive toxicity in humans and animals, raise many concerns because infertility has a significant physical and emotional impact on the decision to use this medicine, particularly among young people (Rezvanfar et al., 2008; Pavin et al., 2018). Icariin, the main active constituent extracted from the traditional herb Epimedium, has been shown to have anti-oxidative, anti-inflammatory, and anti-apoptotic properties. It is now being considered as a potential therapeutic agent for a wide range of disorders, including reproductive system protection (He et al., 2020).

Based on CP-induced spermatogenesis dysfunction mouse models, we demonstrated that ICA has a testis protection effect. ICA effectively alleviated CP-induced testis injury in mice by regulating the expression of Purine metabolism, the PI3K-Akt signaling pathway, necroptosis, the mTOR signaling pathway, glycophospholipid metabolism, the FoxO signaling pathway, the AMPK signaling pathway, and ABC transporters, as well as reducing the severity of the testis tissue pathology change.

### ICA protects against CP-induced spermatogonia injury in mice

Previous research has shown that CP can harm testicular morphology through oxidative damage (Wang et al., 2021), suppressed spermatogenesis (Özbilgin et al., 2021), decreased sperm quality (Park et al., 2021), and significant seminiferous tubule morphology injury (Zhang et al., 2021). The macroscopic and microscopic changes in the testis in the CP group were consistent with previous findings, including abnormal testicular development, reduced testicular volumes and organ ratios, significantly lower sperm count, and spermatogonia cell ultrastructure damage. However, administration of ICA resulted in a significant reduction in testicular injury caused by CP, as well as an increase in sperm count and spermatogonia structure.

Previous research has suggested that the damaging effect of CP on testicular germ cells may reduce sperm count (Shittu et al., 2019; Hai et al., 2021). In our study, we discovered that the number of abnormal sperm and the rate of sperm deformity increased significantly, which was consistent with previous research findings. Furthermore, when compared to the CP group, ICA could reduce the rate of sperm abnormality and increase sperm count. These findings suggested that ICA could reduce testicular injury caused by CP and protect sperm. To better understand the underlying mechanism, we stained testis tissue with H&E, which revealed that the number of cells in the seminiferous tubules was reduced and large lumens existed. Whereas a higher count of spermatozoa was found in the tubules of CP-treated mice given ICA. Furthermore, TEM results revealed that spermatogonia necrosis and apoptosis were significantly higher in the CP group than in the control and ICA groups. As a result, we hypothesized that ICA could reduce CP-induced testicular changes by inhibiting spermatogonia necrosis and apoptosis.

### Integrated multi-omic analysis revealed the molecular mechanism of the protective efficiency of ICA on CP-induced testicular injury

We identified 1393 differentially expressed proteins in testis tissue from the Ctrl, CP, and ICA groups based on proteome results. We discovered that these proteins are involved in a variety of biological processes, including monosaccharide transport and metabolism, nucleotide biosynthetic process, monosaccharide metabolic process, ligase activity, and ATP-dependent activity, all of which are required for normal physiological functions in cells. Furthermore, metabonomics findings show that the differential metabolites from three groups were significantly enriched in Sphingolipid metabolism, Alanine, aspartate, and glutamate metabolism, Glutathione metabolism, and Arginine and proline metabolism pathways. To identify the key pathway of ICA in the treatment of spermatogenesis dysfunction in mice, we examined the most significantly enriched pathways in the proteome and metabolome, which included the PI3K-Akt signaling pathway, necroptosis, the mTOR signaling pathway, glycophospholipid metabolism, and ABC transporters.

Cell survival, growth, proliferation, angiogenesis, transcription, translation, and metabolism are all regulated by the PI3K-Akt/mTOR signaling pathway (Ediriweera et al., 2019; Tewari, Patni, Bishayee, Sah, Bishayee). The dysregulated PI3K-Akt/mTOR signaling pathway has been reported as the most frequently altered signaling pathway in male infertility, making it one of the most important signaling pathways for therapeutic intervention in male infertility (Wu et al., 2020; Long et al., 2021). HSP90, PP2A, Casp9, mTOR, SLC7A5, LPR5/6, ERK1/2, and Grb10 protein levels were activated in CP-induced spermatogenesis dysfunction testes, which were required for PI3K-Akt/mTOR activation. However, ICA effectively modified these key markers of the PI3K-Akt/mTOR pathway, causing them to return to normal levels in the testes after ICA treatment.

Necroptosis is a cellular response to environmental stress that can be caused by chemical and mechanical injury, inflammation, or infection. It differs from apoptosis in several ways, and previous research has linked it to a variety of diseases (Gong et al., 2019; Khoury et al., 2020). The activation of HSP90 and USP21, which leads to the activation of receptor-interacting protein kinase 1 (RIPK1), can cause necroptosis (Molnár et al., 2019). ICA inhibited RIPK1 by suppressing the expression of HSP90 and USP21, and it effectively blocked necroptosis in spermatogonia. ABC transporters are primary transporters found in all domains of life that couple the energy stored in ATP to the translocation of divese molecules across the membrane (Beis, 2015). ABCB1, ABCB8, ABCB11, and ABCG2 transporters protect various organs from toxic insult (Locher, 2016). CP significantly reduced the expression levels of ABCB1, ABCB8, ABCB9, ABCB10, and ABCB11. ICA increased the proten levels of these transporters, preventing testicular spermatogonia cell injury. Glycerophospholipids are the most common type of lipid found in mammalian cell membranes and play an important role in cellular functions such as signal transduction and protein function (Locher, 2016). Furthermore, CP disrupts the glycerophospholipids metabolism pathway in the testis, whereas ICA significantly promotes the addition of glycerophospholipids metabolism. Furthermore, enrichment analysis of metabolic and proteic pathways revealed that glycerophospholipid metabolism is a potential target metabolic pathway for ICA intervention in CP-induced spermatogenesis dysfunction in mice.

In conclusion, we used proteomic and metabolomic analysis to investigate the pharmacodynamic properties of ICA in alleviating CP-induced acute testis injury. The main finding of this study is that ICA protects against spermatogonia injury by influencing the expression of related proteins in CP-induced spermatogenesis dysfunction in mice *via* the pathways of PI3K-Akt signaling, Necroptosis, mTOR signaling, Glycerophospholipid metabolism, and ABC transporters. These findings support the idea that icariin can help with CP-induced testicular dysfunction. Furthermore, our findings point to novel mechanisms of action for icariin. Our findings are significant for future research into the molecular mechanisms underlying the use of ICA in the prevention of CP-induced testicular dysfunction in men.

## Data Availability

The original contributions presented in the study are publicly available. This data can be found here: https://www.iprox.cn/page/PSV023.html;?url=1670294860248KOpS. Accession number: IPX0005509000.

## Supplementary material

Supplementary Figure S1 Johnsen score of testes in different groups.

Supplementary Figure S2 PCA of proteomic data for each group.

Supplementary Figure S3 The Venn diagram shows the overlap between different protein groups.

Supplementary Figure S4 OPLS-DA score plots. On the left are positive ions, and on the right are negative ions. **(A)** “Ctrl vs. CP”, **(B)** “CP vs. ICA”, **(C)** “Ctrl vs. ICA”.

Supplementary Figure S5 Verification of differentially expressed genes by RT-PCR, **p* < 0.05; ***p* < 0.01; ****p* < 0.001.

Supplementary Table S1 The expression trends of proteins in C4, C6, and C7.

Supplementary Table S2 The enriched GO terms of dysregulated proteins.

Supplementary Table S3 The enriched KEGG pathways of dysregulated proteins.

Supplementary Table S4 The dysregulated metabolites in Ctrl vs. CP.

Supplementary Table S5 The dysregulated metabolites in CP vs. ICA.

Supplementary Table S6 The dysregulated metabolites in Ctrl vs. ICA.

Supplementary Table S7 The KEGG enrichment of differentially expressed metabolites in Ctrl vs. CP.

Supplementary Table S8 The KEGG enrichment of differentially expressed metabolites in CP vs. ICA.

Supplementary Table S9 The KEGG enrichment of differentially expressed metabolites in Ctrl vs. ICA.

Supplementary Table S10 The KEGG enrichment of differentially expressed proteins and metabolites in Ctrl vs. CP.

Supplementary Table S11 The KEGG enrichment of differentially expressed proteins and metabolites in CP vs. ICA.

Supplementary Table S12 The primers used in this study.
