# Supplementary figures and images for: Integrative proteomics and metabolomics approach to identify the key roles of icariin-mediated protective effects against cyclophosphamide-induced spermatogenesis dysfunction in mice
Source: Front Pharmacol. 2022 Dec 14;13:1040544. doi: 10.3389/fphar.2022.1040544 (PMC9794755; doi:10.3389/fphar.2022.1040544)

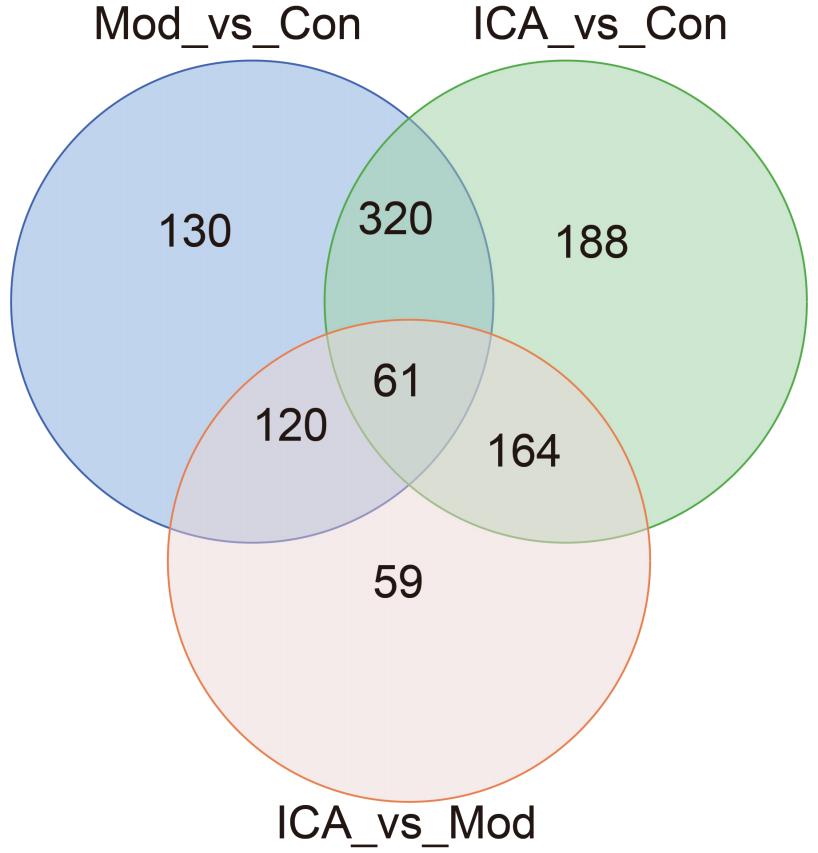

Supplement: Supplementary file 1 [file Image3.JPEG]

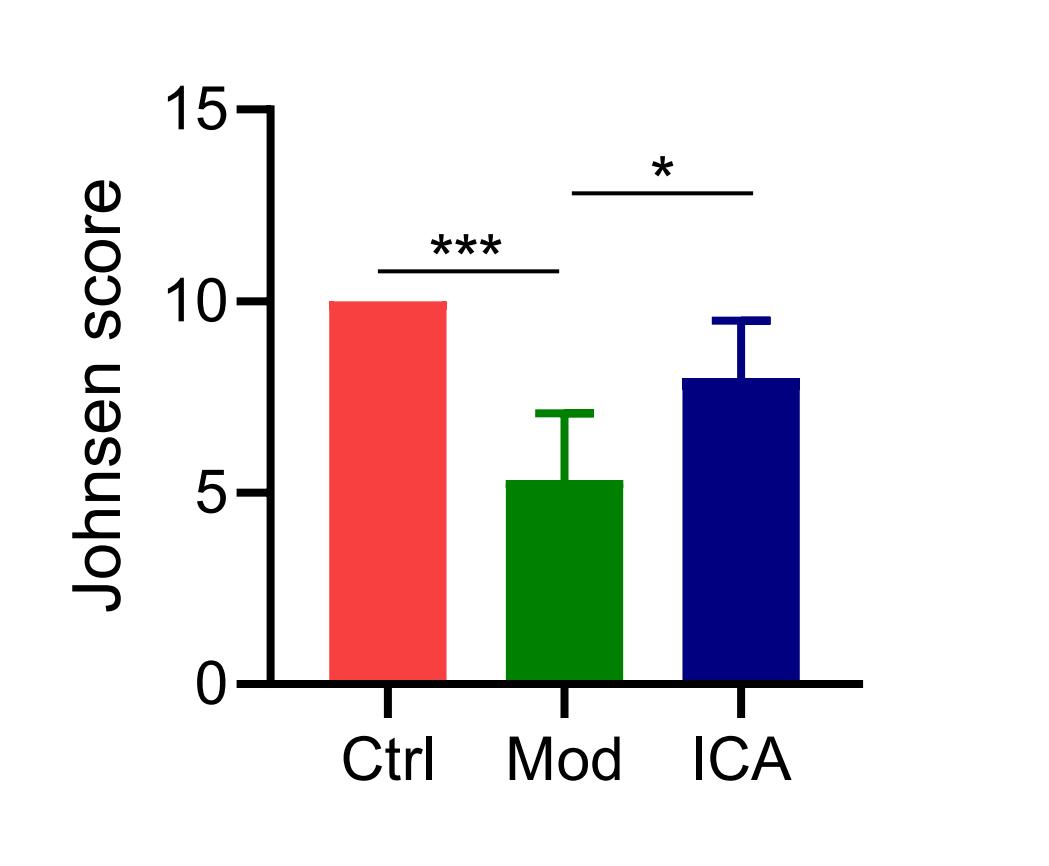

Supplement: Supplementary file 6 [file Image1.JPEG]

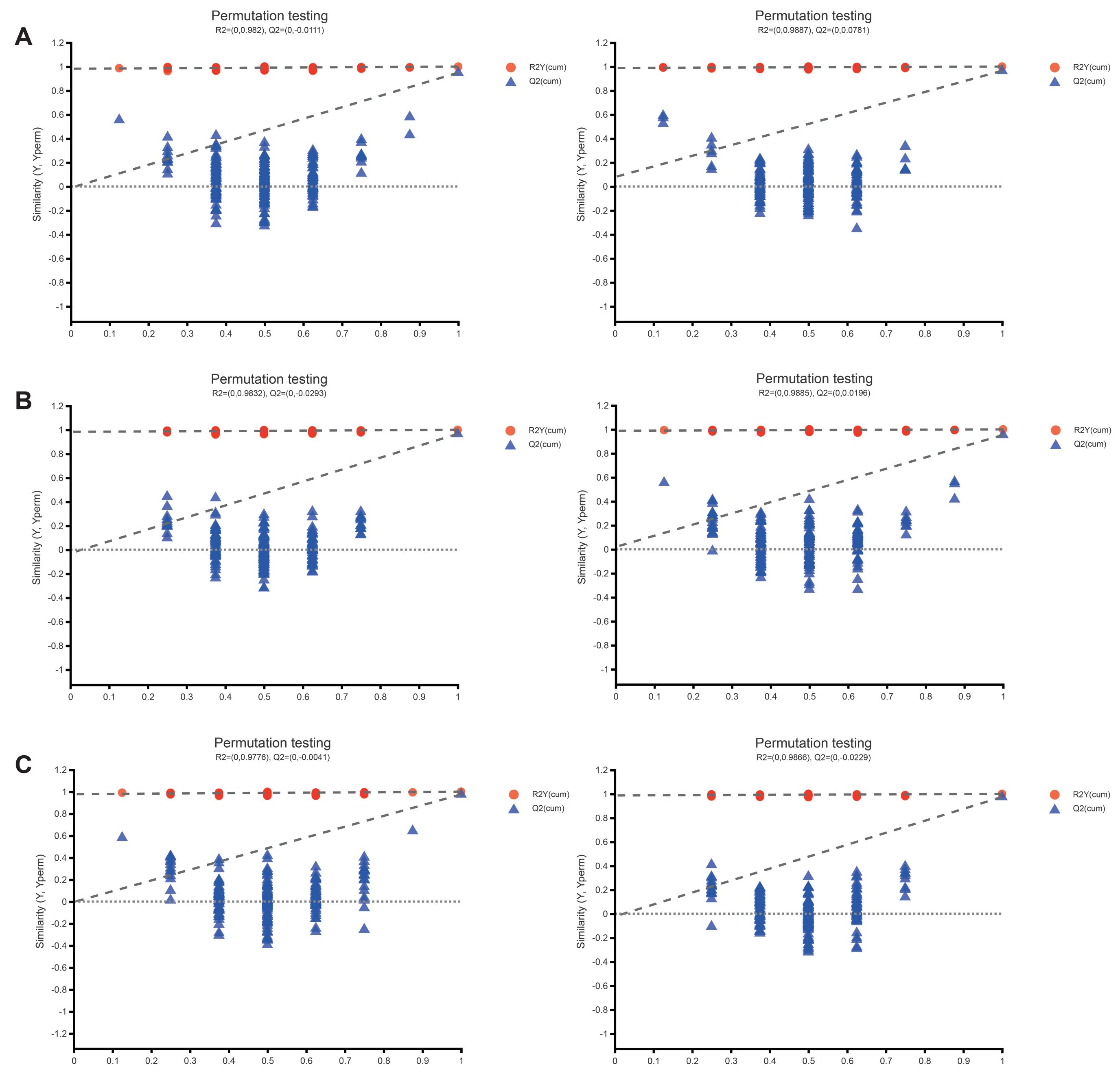

Supplement: Supplementary file 7 [file Image4.JPEG]

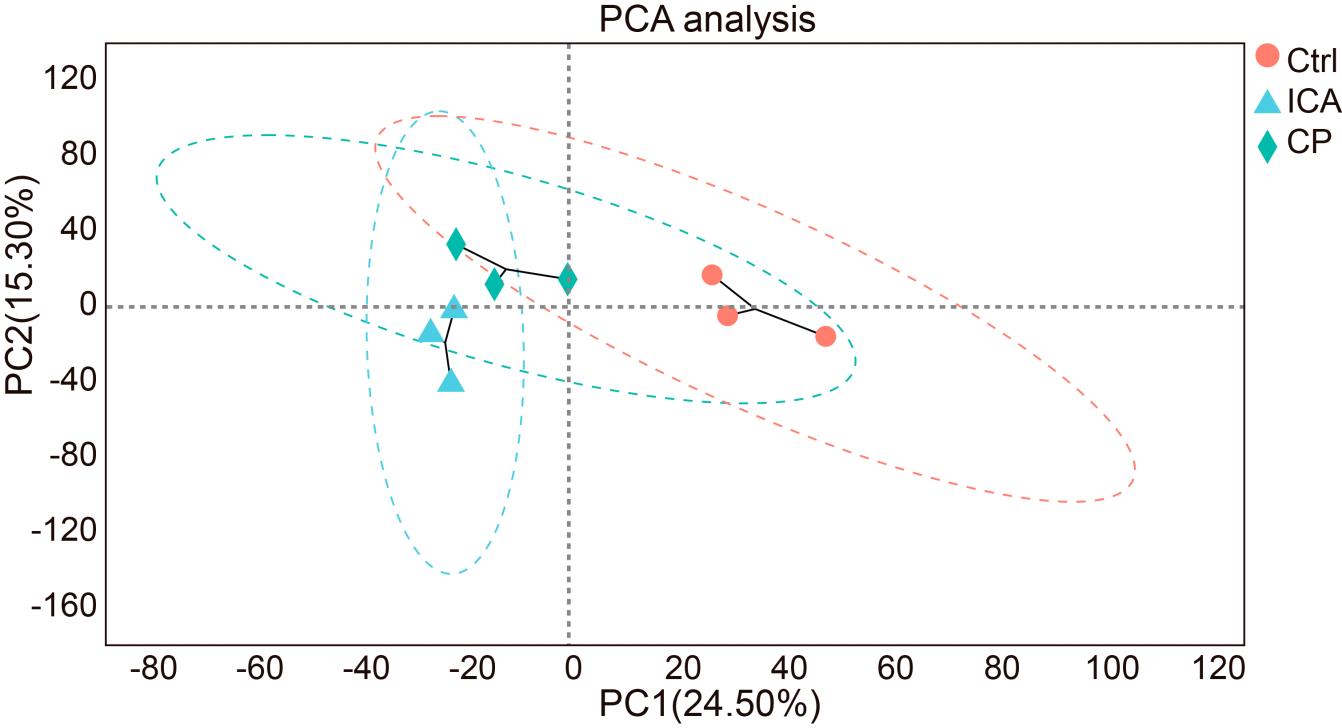

Supplement: Supplementary file 9 [file Image2.JPEG]

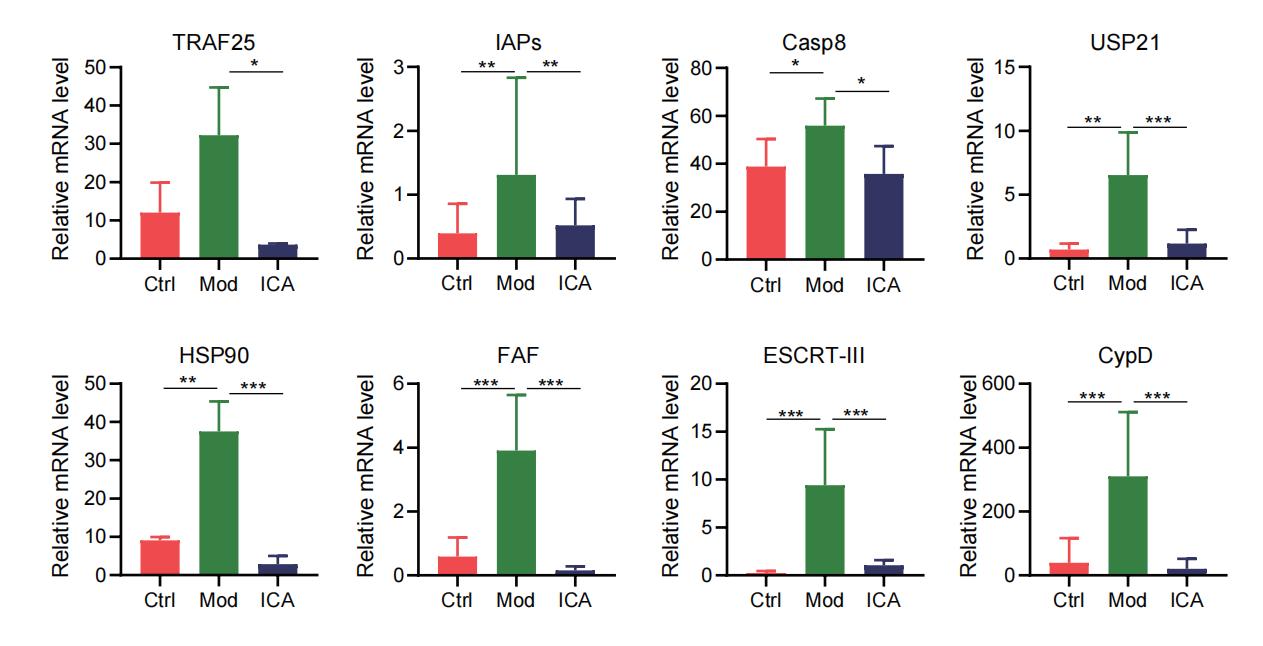

Supplement: Supplementary file 10 [file Image5.JPEG]
